# TCF/LEF Transcription Factors: An Update from the Internet Resources

**DOI:** 10.3390/cancers8070070

**Published:** 2016-07-20

**Authors:** Dusan Hrckulak, Michal Kolar, Hynek Strnad, Vladimir Korinek

**Affiliations:** 1 Department of Cell and Developmental Biology, Institute of Molecular Genetics, Academy of Sciences of the Czech Republic, Videnska 1083, Prague 4 14220, Czech Republic; hrckulak@img.cas.cz; 2 Department of Genomics and Bioinformatics, Institute of Molecular Genetics, Academy of Sciences of the Czech Republic, Videnska 1083, Prague 4 14220, Czech Republic; michal.kolar@img.cas.cz (M.K.); strnad@img.cas.cz (H.S.)

**Keywords:** Wnt signaling, splicing isoforms, GTEx, Fantom5, the Cancer Genome Atlas

## Abstract

T-cell factor/lymphoid enhancer-binding factor (TCF/LEF) proteins (TCFs) from the High Mobility Group (HMG) box family act as the main downstream effectors of the Wnt signaling pathway. The mammalian TCF/LEF family comprises four nuclear factors designated TCF7, LEF1, TCF7L1, and TCF7L2 (also known as TCF1, LEF1, TCF3, and TCF4, respectively). The proteins display common structural features and are often expressed in overlapping patterns implying their redundancy. Such redundancy was indeed observed in gene targeting studies; however, individual family members also exhibit unique features that are not recapitulated by the related proteins. In the present viewpoint, we summarized our current knowledge about the specific features of individual TCFs, namely structural-functional studies, posttranslational modifications, interacting partners, and phenotypes obtained upon gene targeting in the mouse. In addition, we employed several publicly available databases and web tools to evaluate the expression patterns and production of gene-specific isoforms of the TCF/LEF family members in human cells and tissues.

## 1. Introduction

The Wnt signaling pathway is one of the major signaling mechanisms regulating cell-fate decisions during embryogenesis and in adult tissues. Nuclear DNA-binding TCF/LEF proteins and their transcriptional cofactor β-catenin represent the key components of the canonical branch of the pathway. In the absence of the Wnt signal, cytoplasmic β-catenin is bound and phosphorylated at its N-terminus by the β-catenin destruction complex composed of two scaffolding proteins, adenomatous polyposis coli (APC) and axis inhibition protein (Axin), and two serine/threonine kinases, casein kinase 1 alpha (CK1α) and glycogen synthase kinase 3 (GSK3). Phosphorylated β-catenin is tagged for proteasome-mediated degradation by F-box-containing beta-transducin repeat containing (β-TrCP) E3 ubiquitin ligase. Binding of the Wnt ligand to the transmembrane receptor complex that includes Wnt receptor Frizzled (Fzd) and co-receptor low-density lipoprotein receptor-related protein (Lrp) 5/6 leads to the recruitment of Axin to the membrane and functional inactivation of the β-catenin destruction complex. This results in cytoplasmic and nuclear β-catenin accumulation. In the nucleus, β-catenin associates with TCFs to activate transcription of Wnt signaling target genes [1,2,3].

It has been well documented that in *Drosophila* and *Xenopus*, without β-catenin (Drosophila β-catenin is called Armadillo), TCFs repress target gene transcription through their interaction with Groucho/transducin like enhancer of split (Gro/TLE) corepressors [4,5]. Homologous TCFs in mammals behave similarly in respect to the TLE-mediated transcriptional repression [6]. However, despite the fact that these proteins are conserved and partially redundant, they have more complex roles in fine-tuning the cellular response to Wnt signals [7,8]. Great effort has been made to discover the unique functions of individual TCFs. From the historical perspective, LEF1 is perceived as the main transcriptional activator, and TCF7L1—despite its ability to interact with β-catenin—as the pathway repressor [9,10]. However, results for TCF7 and TCF7L2 are contradictory and/or dependent on the particular model system. In addition, all TCFs are produced in multiple isoforms generated by mRNA splicing or alternative promoter usage. Since these isoforms differ in the functional domain composition, they display differences in DNA or cofactor binding, which results in changes in the target gene activation or selection (reviewed in [11]).

## 2. Structural Domains and Posttranslational Modifications of TCFs

**Figure 1:**
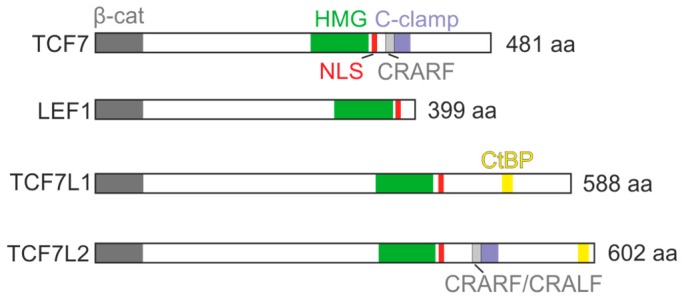
Major functional domains present in TCFs. The diagrams show the position of conserved functional domains in the TCF/LEF family members encoded by Ensemble database transcripts ENST00000395029 (encodes human TCF7), ENST00000265165 (LEF1), ENST00000282111 (TCF7L1), ENST00000627217 (TCF7L2). β-cat, β-catenin-binding domain (gray); HMG, High mobility group-box DNA-binding domain (green); NLS, nuclear localization signal (red); C-clamp, C-clamp DNA-binding motif (blue); CtBP, the domain mediating interaction with the C-terminal binding protein (yellow). The grey bands in the C-clamp motif represent the position of the CRARF/CRALF sequences that contain the first cysteine residue (of four in total) essential for helper sequence binding; aa, amino acids. For the sake of clarity, the size of boxes representing the NLS signal and CRARF/CRALF sequences (in this and other figures) does not correspond to the scale of other depicted domains.

Full-length isoforms of the mammalian TCF/LEF family members contain several functional domains that are conserved in all TCFs identified in various metazoan organisms (see the graphical summary of human TCF/LEF proteins in Figure 1). DNA binding is mediated by a monomeric HMG box domain that recognizes the consensus sequence 5′-ACATCAAAG-3′ called the Wnt response element (WRE) [12,13,14]. The domain contacts DNA through the minor groove of the double helix, which results in DNA bending [15,16]. In the original studies describing functional properties of TCF7 and LEF1, the HMG box was characterized as an approximately 70-amino-acid (aa) domain interacting with the 5′-AACAAAG-3′ sequence motif [17,18,19]. This finding was supported by combinatorial mutagenesis of the WRE [13,20]. Subsequently, TCF7L2 and β-catenin chromatin immunoprecipitation experiments combined with DNA array-based genome-wide analysis or coupled with massively parallel sequencing (ChIP-Seq), respectively, confirmed (and extended) the WRE to its recent form [21,22]. In addition, a short stretch of basic aa in the HMG box C-terminus functions as the nuclear localization signal (NLS) and, moreover, it also stabilizes the interaction with DNA [17,23]. Vertebrate TCF7E and TCF7L2E transcription variants, as well as *Drosophila* and *Xenopus* TCFs, contain an additional (“helper”) DNA-binding domain that seems to have important functional consequences (see below) [14].

TCF7 and LEF1 were originally reported as bona fide transcription activators [9,19]. However, in the present (generally accepted) view, only the β-catenin-TCF complex activates transcription, and establishment of the Wnt-β-catenin-TCF axis was an important discovery in the cellular signaling field. The majority of TCF isoforms possess a conserved N-terminal β-catenin interaction domain. Deletion of this domain abrogates TCF-mediated transcriptional activation and results in developmental defects in *Drosophila* and *Xenopus* embryos [1,12,24]. In human and mouse cells, naturally occurring N-terminal deletion TCF isoforms acts as dominant negative regulators of Wnt signaling [25,26,27]. Interestingly, it has been documented that β-catenin is not the only TCF coactivator and that the activating transcription factor (ATF) family members also activate TCF-driven transcription [28]. In addition, LEF1 was reported to interact with specific partners to drive expression of a distinct set of target genes (addressed below).

In the absence of Wnt signaling, TCFs function as transcriptional repressors [29,30]. In agreement with this model, the Gro/TLE-binding domain, a hydrophobic stretch of aa is present in the central portion of all TCFs [4,5,6]. The interaction is partially supported by the HMG box domain [31]. The repressive mechanism depends on histone deacetylases associated with Gro/TLE [6]. The involvement of TCF-Gro/TLE complexes in the repression of Wnt signaling target genes has been supported by studies in *Drosophila*, *Xenopus* and *C. elegans* [4,32]. Mammalian and *Xenopus* XTCF7L1 and some mammalian TCF7L2 isoforms contain the C-terminal binding protein (CtBP) domain a short aa sequence located in the C-terminal part of the proteins mediating the interaction with CtBP1/2. It is not clear whether CtBP binding exerts any functional effect since contradictory data were shown in different experimental setups. The C-terminal binding proteins were described as short-range transcriptional corepressors (reviewed in [33]); however, the results obtained in *Drosophila* documented that CtBP might act as both activator and repressor of Wnt target genes in a context-dependent manner. Additionally, the CtBP-repressive function was independent of its interaction with TCF [34]. In *Xenopus*, the interaction with CtBP was responsible for TCF7L1-mediated repression of TCF7L1-bound genes [35]. In human cells, CtBP1 binding to TCF7L2 suppressed the activity of the Wnt reporter TOPFlash and expression of the Wnt target gene *AXIN2* [36]. In contrast, in colorectal carcinoma (CRC), CtBP in cooperation with TCF7L2 activated β-catenin-dependent target gene expression and promoted growth and self-renewal of CRC cells [37]. Moreover, Hamada and Bienz showed that CtBP decreased TCF-mediated transcription indirectly by binding to APC. The APC-CtBP complex sequesters free β-catenin, which results in reduced levels of β-catenin-TCF complexes [38].

The TCF activity can also be regulated by several types of post-translational modifications. LEF1, TCF7L1, and TCF7L2 are phosphorylated—upon Wnt signaling activation—by homeodomain-interacting protein kinase 2 (HIPK2) in the HMG box domain. This phosphorylation results in the disruption of TCF binding to DNA [39,40]. Similarly, LEF1 and TCF7L2 DNA binding is abrogated by phosphorylation of their central region by mitogen-activated (MAP) kinase-related nemo like kinase (NLK) [41]. This phosphorylation represents the first step in NLK-related, NLK-associated RING finger protein (NARF)-mediated ubiquitination of TCFs and their subsequent degradation by the proteasome [42]. Additionally, TCF7L2 is phosphorylated on Ser154 by TRAF2 and NCK-interacting kinase (TNIK). The modification increases Wnt target gene activation. Furthermore, TNIK is a part the β-catenin-TCF7L2 complex and the enzyme is overexpressed in intestinal tumors [43,44]. TCF7L2 was reported to be sumoylated on Lys297. Even though this modification does not change its DNA and β-catenin-binding capacity, it enhances the TCF7L2 ability to activate transcription [45]. Finally, in *Drosophila*, the CREB-binding protein acetylates the single TCF homolog, dTCF, at the Armadillo-binding domain, obstructing the protein–protein interaction and suppressing the dTCF function [46].

## 3. Functional Properties and Isoform Expression of Individual TCFs

Recent developments in sequencing technologies enabled analysis of cell genomes and expression profiles in a comprehensive manner. Importantly, many large datasets that include gene expression data of various human tissues including cancer specimens are publicly available. For bioinformatics analysis of TCF/LEF gene expression, transcriptional start site (TSS) usage, and production of splicing isoforms we selected and analyzed data obtained by three different projects/consortia. The Genotype-Tissue Expression project (GTEx; http://www.gtexportal.org) collects distinct human tissues from multiple donors and analyzes mRNA expression in samples imposed on the genomic sequence of the donor to reveal any biological consequence of genetic variability. A related objective of the project is to map and annotate expression quantitative trait loci (eQTLs) in the human genome. In the pilot project, mRNA sequences in 1641 samples of 43 different tissues obtained from 175 individuals were sequenced [47]. We employed the accessible data of the GTEx project to track expression patterns of the TCF/LEF genes in human tissues and to compare the data with the dataset from the functional annotation of the mammalian genome 5 (Fantom5) project (http://fantom.gsc.riken.jp). The Fantom5 project offers the promoter atlas of mammalian genes obtained from 975 human and 399 mouse samples. Active promoters were mapped by cap analysis of gene expression (CAGE) and single-molecule sequencing to reveal TSSs [48]. The project uses methodology that enables measurement of the absolute count of individual transcripts in the analyzed cell type or tissue. The expression levels of genes (or their variants) can be inferred from the information obtained.

### 3.1. TCF7

**Figure 2:**
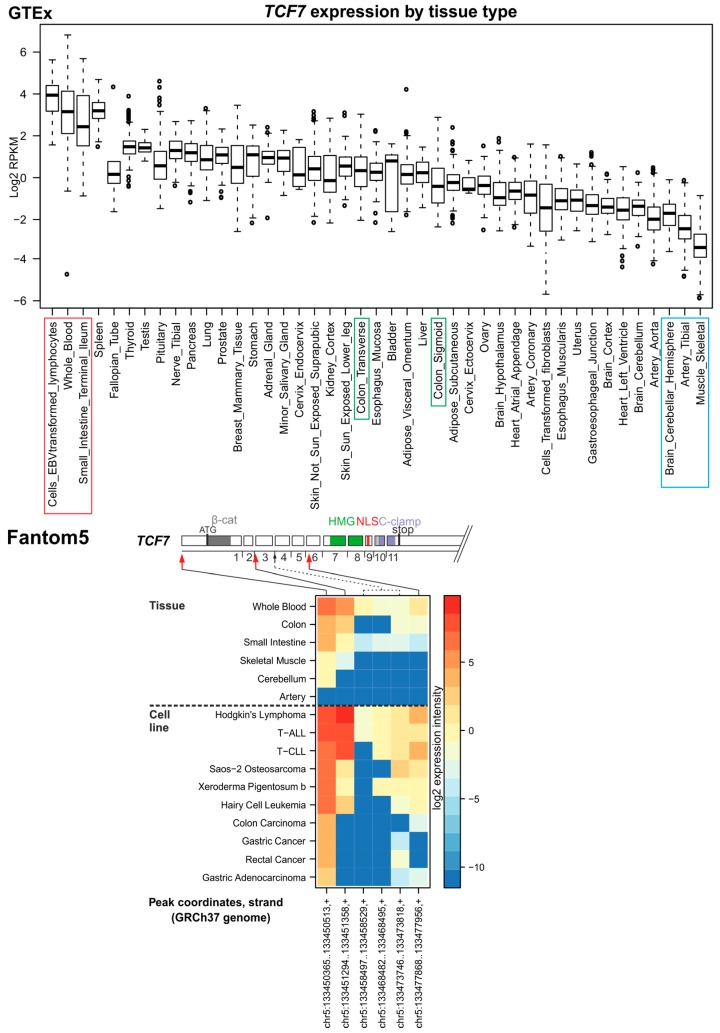
*TCF7* expression in human tissues and TSS usage. Upper panel, expression data obtained from the GTEx portal indicating the *TCF7* expression levels in 43 different adult human tissues. Three tissue types with the highest and lowest *TCF7* expression are boxed in red or blue, respectively. The intestinal specimens are boxed in green, unless they fall into the “high” or “low” expression group. The expression level is given as binary logarithm (log2) of gene-specific reads per gene length (in kilobases) per million of mapped reads (RPKM). The box-and-whiskers plots represent statistical evaluation of results of RNA-seq experiments performed with RNA samples obtained from different individuals. Upper and lower box borders indicate first and third quartiles, respectively. Median is represented by the bold line inside each box. The extent of adjoining values (1.5 interquartile range (IQR)) is depicted by “whiskers”; open circles correspond to outlying samples. Lower panel, mapping TSSs using the Fantom5 project data. Heatmap shows the position of TSSs and their usage as inferred from CAGE. The genome localization of peaks of RNA-seq reads defining individual TSS is indicated below the heatmap; the peaks were also mapped to the gene exon/intron structure (shown above the heatmap). Exon numbers and regions encoding functional domains are depicted (see also Figure 1); for the sake of clarity, the length of introns is not to scale. Red arrows mark peaks (or a group of peaks) representing the major TSSs; a minor TSS is indicated by the black arrow. Putative TSSs located in the last exon and 3′ untranslated region (3′ UTR) were omitted. The analysis was performed with tissue samples corresponding to the boxed specimens in the GTEx diagram (shown above the dashed line in the heatmap) except for the EBV-transformed-lymphocyte sample that is not present in the Fantom5 database. In addition, two colonic tissues in the GETx collection, i.e., transverse and sigmoid colon, are represented by only one colon specimen in Fantom5. Results below the dashed line indicate CAGE analysis of RNA samples isolated from 10 cancer/leukemia cell lines with the highest *TCF7* expression. T-ALL, T-cell acute lymphoblastic leukemia; T-CLL, T-cell chronic lymphocytic leukemia. The methodology is described in detail in Supplementary Materials.

During mouse embryogenesis, the *Tcf7* gene is expressed in a complex pattern partially overlapping with Lef1. In the adult tissue, high Tcf7 expression levels are found mainly in cells of lymphoid lineages [49,50]. Tcf7 knockout animals are viable and fertile; however, they display block in T-lymphocyte differentiation. The relatively mild phenotype implies that the Tcf7 activity is (partially) substituted by other TCFs [51]. Indeed, *Tcf7*/*Lef1* double knockout animals display severe phenotype [8]. Similarly, Tcf7l2 and Tcf7 compound null mice show defects in hindgut expansion and severe caudal truncations [52]. Adult Tcf7 null animals spontaneously develop adenomas of the mammary gland and intestine indicating the tumor suppressive function of the protein [53]. The adenomas display strong nuclear β-catenin positivity and phenotypically resemble tumors in APC^Min^ (“Min” stands for *multiple intestinal neoplasia*) mice. Of note, the mice contain only one functional allele of the *Apc* gene and throughout their life develop multiple Apc-deficient polyps localized mainly to the small intestine and colon (reviewed in [54]). *Tcf7* expression in the healthy mouse intestine is mostly confined to lymphoid cells, but low *Tcf7* expression levels were also detected in epithelial cells lining the bottom part of the intestinal crypt. However, the Tcf7 production is remarkably upregulated in intestinal adenomas [55]. Analysis of expression data from the GTEx database confirmed high *TCF7* expression levels in blood cells, spleen and the small intestine, and (relatively) low expression in the central nervous system, heart, arteries and skeletal muscle (Figure 2, upper panel).

The mouse *Tcf7* gene contains two alternative promoters driving expression of the full-length Tcf7 or N-terminally truncated dominant negative (dn) isoform lacking the β-catenin interaction domain [56]. Both promoters are possibly activated by an adjacent enhancer containing TCF-binding sites, implying a regulatory feed-back loop [53]. TCF7 knock-down experiments in HCT116 and DLD-1 CRC cells displaying hyperactive Wnt signaling indicated that the protein mainly functions as a positive Wnt signaling regulator [57]. The human *TCF7* gene (similarly to the mouse gene) contains two promoters [56]. According to the NCBI RNA reference sequence collection (RefSeq), the *TCF7* (alias *TCF7*) gene gives rise to five transcribed isoforms. However, the Ensembl database indicates up to 14 protein-coding isoforms, although the majority of them are poorly supported by experimental evidence. The evaluation based upon the Fantom5 TSS annotation revealed that both of these promoters are used in tissues with high or low *TCF7* expression or in cell lines derived from leukemia or solid tumors (Figure 2, lower panel; the first two major peaks indicated by red arrows). We attempted to quantify whether in some specimens there is a clear preference for particular promoter usage; however, due to the relatively low number of samples the results were insignificant. Interestingly, we noted an alternative TSS located in intron 6 of the gene; nevertheless, experimental evidence supporting the existence (and function) of the corresponding truncated TCF7 form is missing.

Interestingly, alternative TCF7 splice variants that encode the N-terminal β-catenin interaction domain but differ in the exons encoding the C-terminal part of the protein display identical capacity to activate transcription from a β-catenin-TCF-dependent synthetic reporter. In contrast, some reporters based on the endogenous regulatory elements might be activated only by the TCF7E (or TCF7L2E, see further) isoform containing the conserved 30-aa sequence present in the “E-tail” [58]. The sequence called the “C-clamp”, due to its relatively high cysteine residue content, represents an additional DNA-interacting domain [59,60]. The domain targets the GC-rich “helper” sequence (5′-RCCGCCR-3′ or 5′-YGGCGGY-3′) placed either upstream or downstream to WREs in the promoter of some Wnt target genes [61,62]. Binding to the helper site presumably stabilizes the interaction of TCF7E to promoters containing weak WREs. Nevertheless, whether the observed (selective) responsiveness of some promoters to the “E” variants depends on the presence of the helper site(s) remains to be determined.

### 3.2. LEF1

**Figure 3:**
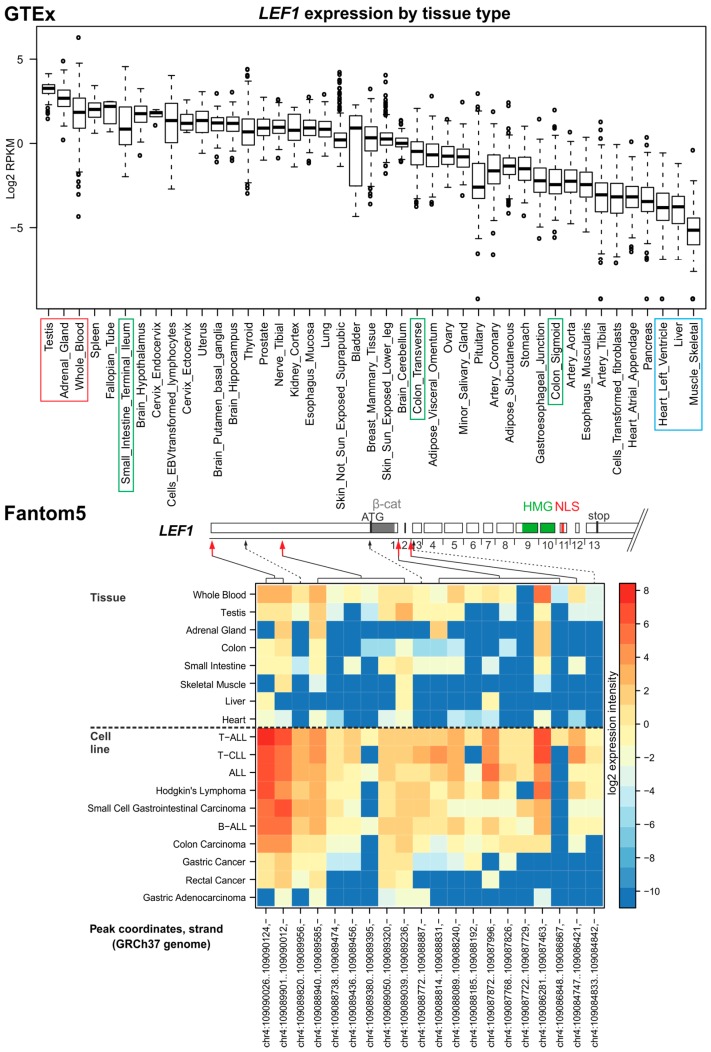
*LEF1* expression in human tissues and TSS usage. For description, see Figure 2 legend. ALL, acute lymphoid leukemia; B-ALL, B-cell ALL.

Similar to Tcf7, Lef1 is widely expressed during mouse embryonic development; however, in adult tissues its expression is tissue-specific, localized mainly to the thymus or T-cells [7,18,50]. Lef1-deficient mice died shortly after birth due to the impaired development of so-called skin appendages including hair, teeth and mammary glands [7]. In the adult mouse intestine, Lef1 expression is mainly restricted to the lymphoid lineage and is not detected in the intestinal epithelium. In contrast, LEF1 is upregulated in intestinal neoplasia, including CRC cells derived from human malignancies [25,55]. The data retrieved from the GTEx database indicate high *LEF1* mRNA levels in the testis, adrenal gland, blood cells, spleen, and ileum. The expression in the latter organ is possibly related to the LEF1 production in lymphoid cells. Substantially lower expression was noted in the heart, arteries, skeletal muscle, pancreas, and liver (Figure 3, upper panel). In human and mouse cells, *LEF1* mRNA is produced from two alternative promoters. Interestingly, a full-length LEF1 isoform that interacts with β-catenin is regulated by β-catenin-TCF complexes. Since constitutive activation of Wnt signaling is a hallmark of the majority of CRC, the observation implies a positive feed-back loop for Wnt signaling favoring expression of the β-catenin-sensitive LEF1 variant in cancer. Additionally, the promoter driving full-length LEF1 includes—next to a WRE—the helper sequence and is preferentially activated by the TCF7E and TCF7L2E isoforms [14]. In contrast, the second promoter located in the second intron of the gene drives expression of dnLEF1 lacking the β-catenin interaction domain [25]. The data suggest a complex regulatory network including feedback loops functioning at the level of TCF-mediated transcription. According to the RefSeq database, there are four alternative isoforms of LEF1 produced in human cells. In the Ensembl database, there are two other protein-coding isoforms to be found, however, they are less well characterized and poorly supported by experimental data. Fantom5 data revealed two (major) TSSs driving production of full-length LEF1 and, in addition, two TSSs located in the first or second intron possibly initiating transcripts encoding dnLEF1. Interestingly, peaks indicating TSS in the first intron were dispersed over a broad genomic region (>1800 nucleotides). Moreover, the data showed high LEF1 levels in leukemic and tumor cells (Figure 3, bottom panel). Subsequent analysis revealed a slight preference for usage of intronic TSSs over TSSs for full-length *LEF1* mRNA in whole blood samples. Strikingly, the preference was reversed in leukemia and lymphoma cells. In contrast, statistical analysis (not shown) did not confirm preferential usage of TSSs for full-length LEF1 in CRC cells.

Apart from its role in Wnt/β-catenin signaling, LEF1 forms transcriptional complexes with ALY (Ally of AML-1 and LEF-1) to activate T-cell receptor α (TCRα) enhancer in a β-catenin-independent manner [63]. Furthermore, some melanocyte-specific genes are regulated by microphthalmia-associated transcription factor (MITF) through its interaction with LEF1 [64].

### 3.3. TCF7L1

Tcf7l1 is the most abundant and crucial member of the TCF/LEF family expressed in early mouse embryos. Tcf7l1-deficient embryos develop expanded (or duplicated) mesoderm structures, including nodes, and do not survive embryonic day 11.5 (E11.5) [10]. Although data from *Xenopus* suggested that TCF7L1 functions as a repressor [29,35,65], Merrill and colleagues did not observe any ectopic activity of the β-catenin-TCF-dependent TOPGal reporter in Tcf7l1^−/−^ embryos. This implied that Tcf7l1 might restrict expression of target genes that are not regulated by β-catenin-TCF complexes [10]. In the mouse epidermis, the absence of Tcf7l1-mediated repression blocks the hair follicle, epidermal, and sebaceous gland programs. Surprisingly, the latter two programs do not involve canonical Wnt signaling [66]. Interestingly, in the skin, Tcf7l1 is redundant with Tcf7l2 since the absence of the single gene had no obvious phenotypic consequences. However, concomitant deletion of both genes resulted in loss of long-term tissue maintenance and in hair follicle growth suppression. Strikingly, conditional ablation of the Wnt mediator β-catenin in the skin induced only partly overlapping phenotype. The majority of genes in Tcf7l1/4-null tissues were upregulated when compared to control cells; moreover, their expression was not influenced by β-catenin inactivation [67]. In *Xenopus* cells, β-catenin binding is required for TCF7L1 phosphorylation and its subsequent dissociation from target promoters upon Wnt signaling activation [39]. This phosphorylation is crucial in the process of exchanging suppressive β-catenin-TCF7L1 to stimulating β-catenin-TCF7 complexes at the promoter sites [40]. A similar “mode of action” was proposed for Tcf7l1/β-catenin in embryonic stem (ES) cells in which Tcf7l1 represses self-renewal and pluripotency genes [68,69]. However, more detailed ChIP experiments showed that Tcf7l1-bound promoters are—upon β-catenin stabilization—activated through a TCF-independent mechanism, presumably via the β-catenin-Oct4 complex [70,71]. In human breast cancer, upon β-catenin binding TCF7L1 dissociates from the chromatin and is degraded by the proteasome, albeit in a phosphorylation-independent manner [72]. Finally, in reporter assays, ectopically expressed TCF7L1 and β-catenin activate transcription from the Wnt reporter TOPFlash [1,73]. In summary, these data are consistent with a model in which TCF7L1 represses genes when β-catenin is absent. Nevertheless, the TCF7L1 Wnt-dependent and Wnt-independent roles remain to be delineated.

**Figure 4:**
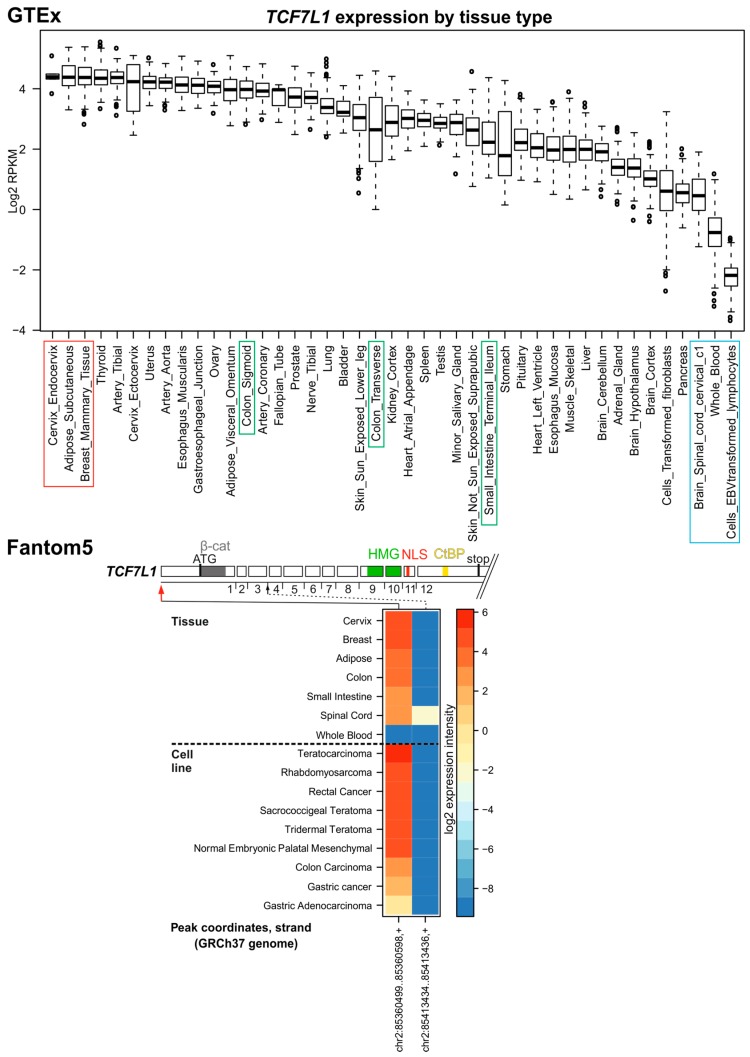
*TCF7L1* expression in human tissues and TSS usage. For description, see Figure 2 legend.

In healthy adult mice, Tcf7l1 is expressed moderately in the colonic crypt compartment [55]. Its expression was also reported in some CRC cell lines [6]. Surprisingly, both GTEx and Fantom5 data revealed relatively high TCF7L1 production in many adult organs including the cervix, breast, colon, adipose tissue, and in CRC cells (Figure 4). TCF7L1 might play a role in CRC progression as sequencing studies revealed recurrent translocations resulting in production of a *neuron navigator 2* (*NAV2*)*-TCF7L1* fusion transcript. The predicted NAV2-TCF7L1 fusion protein lacks the β-catenin binding domain [74]. According to the RefSeq database, only one major TCF7L1 isoform is expressed in human cells. The variant contains the E-tail, but its sequence is less conserved and lacks the C-clamp domain. The C-terminal part of the protein interacts with CtBP corepressor, but the functional meaning of this interaction has never been tested in vivo [35]. Accordingly, analysis of Fantom5 data showed only a single TSS initiating the full-length TCF7L1 transcript. Additionally, no splice variants occurring in the healthy colon or CRC specimens were detected.

### 3.4. TCF7L2

**Figure 5:**
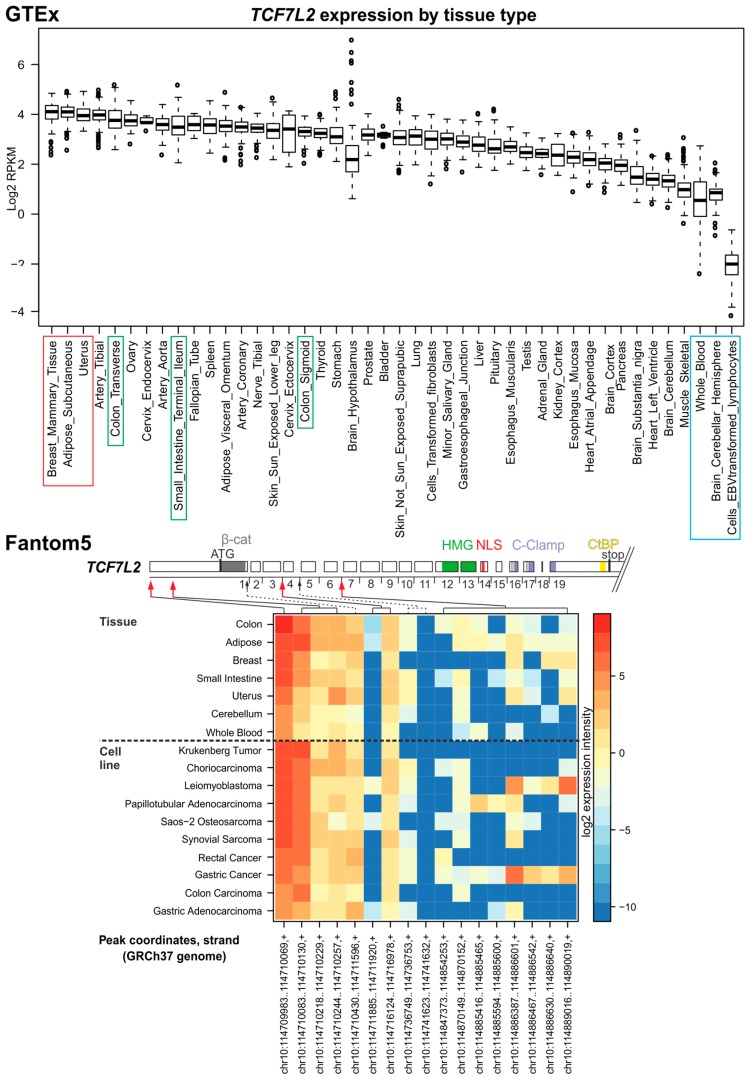
*TCF7L2* expression in human tissues and TSS usage. For description, see Figure 2 legend.

TCF7L2 is the most intensely studied member of the TCF/LEF family. During mouse embryogenesis, its expression is the highest in the gut and central nervous system. However, in adult tissues, the gene is produced in a more ubiquitous pattern [75]. Moreover, the gene is highly expressed in CRC-derived cells [75]. This is in agreement with the GTEx and Fantom5 data showing rather uniform TCF7L2 expression in many adult tissues. Moreover, TCF7L2 displays—among the TCF/LEF family members—the highest expression in the intestine and colon. In contrast to TCF7 and LEF1, TCF7L2 (and TCF7L1) is less abundant in blood cells (Figure 5).

TCF7L2 is crucial for proper development of the small intestinal and colonic epithelium tissue homeostasis in the adult intestine [76,77,78]. Nevertheless, the gene targeting experiments also brought several conflicting results. “Classical” whole-body Tcf7l2 knockout resulted in neonatal death. The most notable phenotype was the absence of the proliferative compartment in the small intestine; however, colonic development seemed to be intact [76]. Conditional inactivation of Tcf7l2 in the adult intestinal epithelium using animals with the floxed genomic sequence encoding the HMG box impaired cell proliferation in both the small intestine and colon [78]. The defect resembled tissue damage observed upon ectopic expression of extracellular Wnt pathway inhibitor Dickkopf1 (Dkk1) [79]. Finally, ablation of Tcf7l2 via Cre recombinase-mediated deletion of the first exon in embryos provoked necrosis of epithelial cells followed by disruption of the small intestinal and colonic architecture at E14.5. Strikingly, in E13.5 embryos, i.e., in embryos just at the beginning of the conversion of pseudostratified undifferentiated endoderm into columnar epithelium, loss of Tcf7l2 provoked increased proliferation of epithelial progenitors [77]. However, in the adult colon, Tcf7l2 deletion resulted in enlarged crypts and, furthermore, Tcf7l2 haploinsufficiency promoted formation of colonic tumors in Apc^Min^, indicating the tumor-suppressive role of Tcf7l2 [77]. Loss-of-function TCF7L2 mutations were found in sporadic CRC, further confirming that apart from its physiological role in healthy intestine, the TCF7L2 status is important for initiation and/or progression of the disease [74]. In addition, a genome-wide RNA-mediated interference (RNAi) screen identified TCF7L2 as a transcriptional repressor decreasing the Wnt pathway output and restricting CRC cell growth [57]. How are the discrepant phenotypes obtained upon *Tcf7l2* gene loss? How is the diminished proliferation in the Tcf7l2-deficient epithelium related to the possible tumor-suppressive role of the factor? These questions still remain to be answered. Since transcription from the targeted alleles is substantially reduced, the alternative that the modified alleles produce aberrant Tcf7l2 protein interfering with the function(s) of additional TCFs can be excluded [77]. In the adult human and mouse colonic epithelium, Tcf7l2 expression is the highest in differentiated cells and Apc loss drives aberrant expression of Lef1 and Tcf7 proteins [55,78]. Thus, we might speculate that in this particular example, Tcf7l2 plays both an activatory and inhibitory role and is replaced by the Wnt pathway activators Lef1 or Tcf7 in the transformed epithelia.

*Xenopus* and mouse Tcf7l2 is produced as an N-terminally truncated variant lacking the β-catenin interaction domain. Similarly to Tcf7 and Lef1 proteins, the variant—initiated from an alternative promoter located in the fifth exon of the mouse gene—blocks transcription from the β-catenin-TCF-dependent promoter [27]. In the Fantom5 data, alternative TSSs/promoters located in introns 3 and 6 that initiate expression of truncated versions of TCF7L2 in human tissue were also detected; nevertheless, their usage seems to be less significant (Figure 5). Additionally, TCF7L2 is expressed in multiple isoforms possibly arising by alternative splicing of TCF7L2 pre-mRNA. The TCF7L2E variant contains the C-clamp motif and similarly to TCF7E, only this variant can activate a specific set of target promoters [14,58,80].

According to the RefSeq or Ensembl database, there are 13 and 18 expressed protein-coding TCF7L2 isoforms in human cells, respectively. Moreover, frameshift mutations in the *TCF7L2* gene resulting in protein truncation occur frequently in tumors showing microsatellite instability (MIS) [81,82]. Moreover, splice site mutations in the *TCF7L2* locus were also reported in CRC [83]. In order to characterize the effect of alternative splice variants on target gene expression, Weise and colleagues cloned 13 different *Tcf7l2* transcripts from mouse cells making up groups of E, S and M variants [58]. The E, S and M variants differ mostly by the exon composition encoding the C-terminus. The E forms contain the complete E-tail with the C-clamp motif, S variants lack the terminal part of the C-clamp and M variants end in exon 15 lacking the whole E-tail. Moreover, members of each group varied in the composition of N-terminal exons. Representative splice variants were used in luciferase reporter assays using promoter sequences of known TCF/LEF target genes. The assay showed that the E-tail containing variants that lack exon 4 exert the greatest capacity to activate transcription of target genes in contrast to rather inactive S and M isoforms [58]. Higher activatory potential of the E-tail variants in comparison to M isoforms was also confirmed in similar experiments by Wallmen and colleagues [80]. However, further analysis is needed to confirm these results since ectopic expression does not entirely reflect the function of endogenously produced proteins.

## 4. In Silico Analysis of TCF/LEF Isoform Expression in the Healthy Colon and CRC Specimens

**Figure 6:**
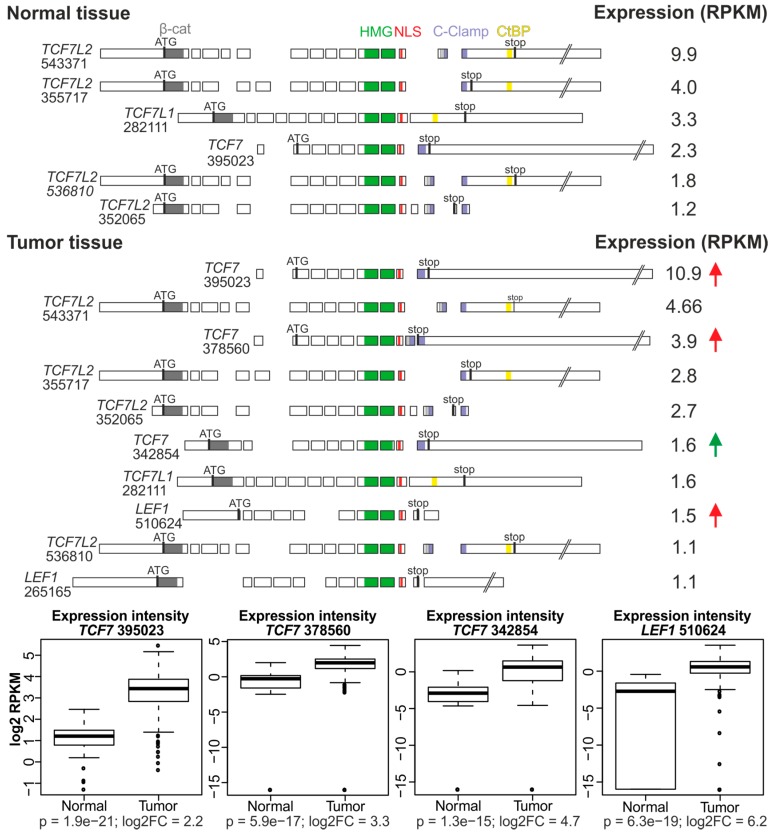
Expression of TCF/LEF isoforms in CRC specimens and normal colonic mucosa analyzed in the TCGA dataset. Upper schemes, a panel of the most abundant TCF/LEF transcripts produced in CRCs and in healthy human colon were sorted by expression intensity indicated as reads per kilobase per million mapped reads (RPKM) as inferred from the RNA-seq experiment; transcripts with RPKM >1 and annotated in the Ensembl genome version GRCh37 are shown. Each transcript is labeled by the last six-digit code from the corresponding Ensembl identificator. Transcripts that are expressed with significantly different abundance in tumor tissue (criterion: binary logarithm of fold change in the expression level (log2 FC) > 2 (*p* < 0.001), nonparametric Mann-Whitney U test) are marked by red (dominant-negative forms) or green (β-catenin-binding forms) arrow. Bottom diagrams, expression intensity box-and-whiskers plots for four TCF/LEF transcripts that are significantly more abundant in tumors compared to healthy colon tissue. The expression level of each selected transcript is statistically evaluated in all normal and tumor samples and represented by box plots. The expression level is given in log2 RPKM. Upper and lower borders of the box represent first and third quartiles, respectively. Median of expression is indicated by the bold line inside each box. The range of adjoining values (1.5 IQR) is shown by whiskers; outlying values are depicted as open circles; the corresponding p value and log2 FC for each transcript is shown at the bottom of each plot. The methodology is described in detail in Supplementary Materials.

The intestinal epithelia of the small intestine and colon represent a suitable model system to study stem cell-based homeostasis in the adult organism. The single-layer epithelium consists of several functionally and morphologically distinct differentiated cell types that are subjected to rapid turnover. The continuous repopulation of epithelia (the rate of the cell renewal is 3–5 days) is maintained by multipotent intestinal stem cells (ISCs) that reside at the bottom of microscopic invaginations called crypts. Specific features of ISCs, such as multipotency, self-renewal, and proliferative capacity, are tightly connected to active canonical Wnt signaling that is locally restricted to the lower part of the crypt (reviewed in [84,85]). Blocking of the Wnt pathway in the intestine diminishes the crypt compartment and disrupts the tissue architecture [76,78,86,87]. Conversely, deregulation of the Wnt/β-catenin pathway in the intestine is invariantly connected to CRC. Recently, several genome-wide studies brought an unprecedented insight into the mutational landscape, epigenetic changes and expression profiles of many different neoplasia including CRC [74,83,88,89]. The Cancer Genome Atlas (TCGA), collaborative project of the National Cancer Institute and National Human Genome Research Institute, provides data from tumors and matching healthy tissue. The current datasets acquired by high-throughput DNA sequencing, RNA-seq, and DNA microarrays include information from 11,000 cancer specimens derived from 33 cancer types. The data are available through the TCGA data portals [90]. From the TCGA datasets we employed RNA-seq results from 287 CRC and from 41 healthy colonic mucosa [74]. We characterized frequencies of various TCF/LEF isoforms in CRCs in comparison to healthy tissue. In normal tissue, the most abundant isoforms were represented by the TCF7L2 and TCF7L1 factors containing the β-catenin interaction domain, and one dnTCF7 isoform missing the C-clamp sequence. In contrast, all TCF7L2 variants included the C-clamp, implying that they bind promoters with the helper element. Furthermore, none of the *TCF7L2* isoforms contained intron 7 and, moreover, exons 4 and 6 were absent in some mRNAs. In tumors, the same *TCF7L2*, *TCF7L1* and *TCF7* isoforms were detected (as in healthy tissue); however, the most remarkable difference was an increase in the expression levels of dnTCFs, including two dnTCF7 variants (one with and one without the C-clamp motif) and one dnLEF1. Subsequent statistical analysis revealed that upregulation of these variants is significant (Figure 6). Nevertheless, since we also observed increased expression of *TCF7* mRNA encoding the activatory form of the protein, the influence of the “cocktail” of different TCF types on the Wnt signaling status in cancer or healthy cells is difficult to assess. Nevertheless, these data clearly point out the unique, possibly indispensable role of TCF7L2 in the healthy intestine.

In summary, in this review article we recapitulated results describing the features, expression patterns, and isoform production of TCF/LEF factors and their phenotypes obtained upon *TCF/LEF* gene inactivation in the mouse. In addition, we employed GTEx, Fantom5, and TCGA datasets and corresponding bioinformatics tools to compare the published expression data with in silico analysis of human samples. The samples included mRNAs isolated from healthy adult tissues, cancer and leukemia cell lines, and CRC specimens. The latter comparison confirmed rather restricted *TCF7* expression pattern and broader *LEF1* and TCF7L2 production across many tissue and cell types. Furthermore, relatively high mRNA levels in a number of specimens were also detected for *TCF7L1*. This was rather unexpected since in the mouse model, *Tcf7l1* expression was predominantly detected during early stages of embryonic development. Moreover, production of multiple TCF/LEF isoforms generated either by alternative promoter usage or mRNA splicing was found in various healthy tissues, cells or CRCs. Strikingly, increased abundance of *TCF7* and *LEF1* isoforms encoding dominant-negative proteins was observed in cancer specimens. The direct impact of the changes in the intracellular composition of TCF/LEF isoforms on the Wnt signaling pathway output remains to be determined.

## Supplementary Materials

The supplementary materials are available online at www.mdpi.com/2072-6694/8/7/70/s1.
