# Supplementary material for: TCF/LEF Transcription Factors: An Update from the Internet Resources
Source: Cancers (Basel). 2016 Jul 20;8(7):70. doi: 10.3390/cancers8070070 (PMC4963812; doi:10.3390/cancers8070070)
Supplement: Supplementary file 1 [file cancers-08-00070-s001.pdf]

Supplementary File

Data acquisition and processing

TCGA

We downloaded the Cancer Genome Atlas data for CRC [1] from the Broad Institute using the utility firehose_get (http://gdac.broadinstitute.org, Broad Institute/Massachusetts Institute of Technology, USA) on October 20, 2015. We extracted the RNA-seq data from the archive "gdac.broadinstitute.org_COAD.Merge__rnaseqv2__illuminahiseq_rnaseqv2__unc_edu__Level_3__ RSEM_isoforms_normalized__data.Level_3.2015060100.0.0" [2] and converted raw read counts to RPKM (reads per million of mapped reads per kilobase of transcript length) using UCSC GRCh37/hg19 genome assembly and gene annotation ([3], table 'knownGene'). Next we evaluated log-expression intensity of a transcript *t* as $I(t) = \log_2(\mathrm{RPKM}(t))$ and relative transcript usage as $U(t) = \mathrm{RPKM}(t) / \Sigma_v \mathrm{RPKM}(v) * 100\%$, where the sum runs over all transcripts *v* of a gene. Finally, we evaluated the changes in expression log-intensity $I(t)$ and usage $U(t)$ between tumor samples ($N = 287$) and normal tissue samples ($N = 41$) using Mann-Whitney U test.

GTEx

We downloaded the Genotype-Tissue Expression Consortium (GTEx) data and metadata from the consortium's portal (http://www.gtexportal.org, file GTEx_Analysis_v6_RNA-seq_RNA-SeQCv1.1.8_gene_rpkm.gct.gz, accessed on February 23, 2016; [4]) and converted the values to log2(RPKM).

FANTOM5

We downloaded the Functional Annotation of the Mammalian Genome consortium (FANTOM5) data and metadata from the consortium's portal (http://fantom.gsc.riken.jp/5/datafiles/latest/ /extra/CAGE__peaks, file hg19.cage_peak_phase1and2combined_tpm_ann.osc.txt.gz, accessed on February 8, 2016; [5]) and extracted tags per million (TPM) values for all peaks overlapping the respective gene coordinates (using ensEMBL GRCh37 genome annotation, [6]). To decrease the dimensionality of the data, we clustered the peaks by their expression similarity over all FANTOM5 samples and over their genomic locations using MCLUST algorithm ([7], default parameters) and labelled the resulting meta-peaks by the minimal and maximal coordinates of the contributing peaks. The meta-peaks consisted of either a single peak or an uninterrupted peak sequence along the genomic axis. In case of *TCF4*, we split one metapeak to two original peaks for better interpretability. The meta-peak TPM was computed as the sum of TPMs of all contributing peaks. For plotting purposes, we averaged expression intensity of the metapeaks in each sample group and transformed the values by base-two-logarithm.

All computations and visualisations of the data were performed within the R environment [8].

Supplementary References

1. Cancer Genome Atlas Network (2012) Comprehensive molecular characterization of human colon and rectal cancer, *Nature.* **487**, 330-7.
2. Li, B. & Dewey, C. N. (2011) RSEM: accurate transcript quantification from RNA-Seq data with or without a reference genome, *Bmc Bioinformatics.* **12**.
3. Speir, M. L., Zweig, A. S., Rosenbloom, K. R., Raney, B. J., Paten, B., Nejad, P., Lee, B. T., Learned, K., Karolchik, D., Hinrichs, A. S., Heitner, S., Harte, R. A., Haeussler, M., Guruvadoo, L., Fujita, P. A., Eisenhart, C., Diekhans, M., Clawson, H., Casper, J., Barber, G. P., Haussler, D., Kuhn, R. M. & Kent,

W. J. (2016) The UCSC Genome Browser database: 2016 update, *Nucleic Acids Research.* **44**, D717-D725.

4. Mele, M., Ferreira, P. G., Reverter, F., DeLuca, D. S., Monlong, J., Sammeth, M., Young, T. R., Goldmann, J. M., Pervouchine, D. D., Sullivan, T. J., Johnson, R., Segre, A. V., Djebali, S., Niarchou, A., Wright, F. A., Lappalainen, T., Calvo, M., Getz, G., Dermitzakis, E. T., Ardlie, K. G., Guigo, R. & Consortium, G. T. (2015) The human transcriptome across tissues and individuals, *Science.* **348**, 660-665.
5. FANTOM Consortium and the RIKEN PMI and CLST (DGT) (2014) A promoter-level mammalian expression atlas, *Nature.* **507**, 462-70.
6. Yates, A., Akanni, W., Amode, M. R., Barrell, D., Billis, K., Carvalho-Silva, D., Cummins, C., Clapham, P., Fitzgerald, S., Gil, L., Giron, C. G., Gordon, L., Hourlier, T., Hunt, S. E., Janacek, S. H., Johnson, N., Juettemann, T., Keenan, S., Lavidas, I., Martin, F. J., Maurel, T., McLaren, W., Murphy, D. N., Nag, R., Nuhn, M., Parker, A., Patricio, M., Pignatelli, M., Rahtz, M., Riat, H. S., Sheppard, D., Taylor, K., Thormann, A., Vullo, A., Wilder, S. P., Zadissa, A., Birney, E., Harrow, J., Muffato, M., Perry, E., Ruffier, M., Spudich, G., Trevanion, S. J., Cunningham, F., Aken, B. L., Zerbino, D. R. & Flicek, P. (2016) Ensembl 2016, *Nucleic Acids Research.* **44**, D710-D716.
7. Fraley, C. & Raftery, A. E. (2002) Model-based clustering, discriminant analysis, and density estimation, *Journal of the American Statistical Association.* **97**, 611-631.
8. R Core Team (2016) R: A language and environment for statistical comupting in *R Foundation for Statistical Computing, Vienna, Austria*
